# Enhancing sepsis therapy: the evolving role of enteral nutrition

**DOI:** 10.3389/fnut.2024.1421632

**Published:** 2024-10-01

**Authors:** Fuchao Xu, Geng Lu, Jun Wang

**Affiliations:** Department of Emergency Medicine, Nanjing Drum Tower Hospital, Nanjing University Medical School, Nanjing, China

**Keywords:** sepsis, enteral nutrition, septic shock, nutritional support, research progress

## Abstract

Sepsis is a life-threatening organ dysfunction syndrome caused by a dysregulated response to infection in the body. Effective treatment of sepsis poses a significant challenge in today’s clinical field. In recent years, enteral nutrition has garnered significant attention as an essential supportive therapeutic strategy. Serving as a means to provide ample nutritional support directly through the gastrointestinal tract, enteral nutrition not only addresses the nutritional depletion caused by the disease but also holds potential advantages in regulating immune function, maintaining intestinal mucosal barrier integrity, and promoting tissue repair. This article delves into the latest advancements of enteral nutrition in the treatment of sepsis, with a particular focus on its application effectiveness in clinical practice, potential mechanisms, and challenges faced. By examining relevant basic and clinical research, the aim is to provide a deeper understanding of nutritional therapy for sepsis patients and offer valuable insights for future research and clinical practice.

## 1 Introduction

Sepsis is a life-threatening syndrome of organ dysfunction caused by a dysregulated host response to infection (1). It poses a significant challenge in the current clinical field. The clinical manifestations of sepsis not only include a systemic inflammatory response but May also be accompanied by multiple organ dysfunction, which necessitates appropriate clinical treatment for the patients (2). In this context, enteral nutrition has garnered increasing attention in recent years as an important supportive treatment strategy.

The importance of the gut in both physiological and pathological processes has been widely recognized. In the development of sepsis, the gut is not only a source of infection but is also considered the “engine” of multiple organ dysfunction syndrome (3). The impairment of gut barrier function and the dysregulation of the immune system make sepsis patients more susceptible to secondary infections and exacerbate systemic inflammation (4, 5). Therefore, targeting the gut has become a breakthrough in alleviating the condition of sepsis patients.

Enteral nutrition, as a means of providing adequate nutritional support directly through the gut, not only compensates for the nutritional depletion caused by the disease but also has potential advantages in regulating immune function, maintaining the gut mucosal barrier, and promoting tissue repair (6). This article will delve into the latest advancements in enteral nutrition in the treatment of sepsis, focusing on its effectiveness in clinical practice, potential mechanisms, and challenges faced. By reviewing relevant basic research and clinical trials, we aim to provide a deeper understanding of nutritional therapy for sepsis patients and offer valuable insights for future research and clinical practice.

## 2 Sepsis and intestinal function

### 2.1 Impact of sepsis on the intestine

Sepsis is a severe systemic infection, with its impact on the gut primarily involving the disruption of the intestinal mucosal barrier due to inflammation. During sepsis, the immune system’s intense response to the infection can lead to the release of inflammatory mediators, which trigger a series of adverse reactions in the intestinal mucosa (2, 7). Firstly, inflammation can increase mucosal permeability, leading to a loosening of the mucosal barrier. Under normal conditions, the intestinal mucosal barrier maintains a relatively tight structure through intercellular junctions and secreted substances to prevent the translocation of harmful substances and microorganisms. However, during sepsis, inflammation-induced changes can cause the mucosal barrier to become more permeable, allowing bacteria, toxins, and other pathogens to traverse the mucosa more easily. Secondly, the inflammatory response can increase vascular permeability, resulting in the leakage of fluids and proteins from the vasculature into the surrounding tissues. These leaked substances can exacerbate damage to the intestinal mucosal barrier and negatively impact normal digestive and absorptive functions. Finally, inflammation-induced cellular damage and cell death can lead to ulceration of the intestinal mucosa. These ulcers are a direct result of mucosal barrier disruption and provide additional entry points for pathogens. Furthermore, the ulcers can cause localized bleeding, worsening tissue damage. Overall, the impact of sepsis on the gut is primarily characterized by inflammation-induced disruption of the intestinal mucosal barrier, leading to increased permeability, fluid leakage, and ulcer formation, which severely interfere with normal gut function.

### 2.2 Role of the immune system

**Figure 1:**
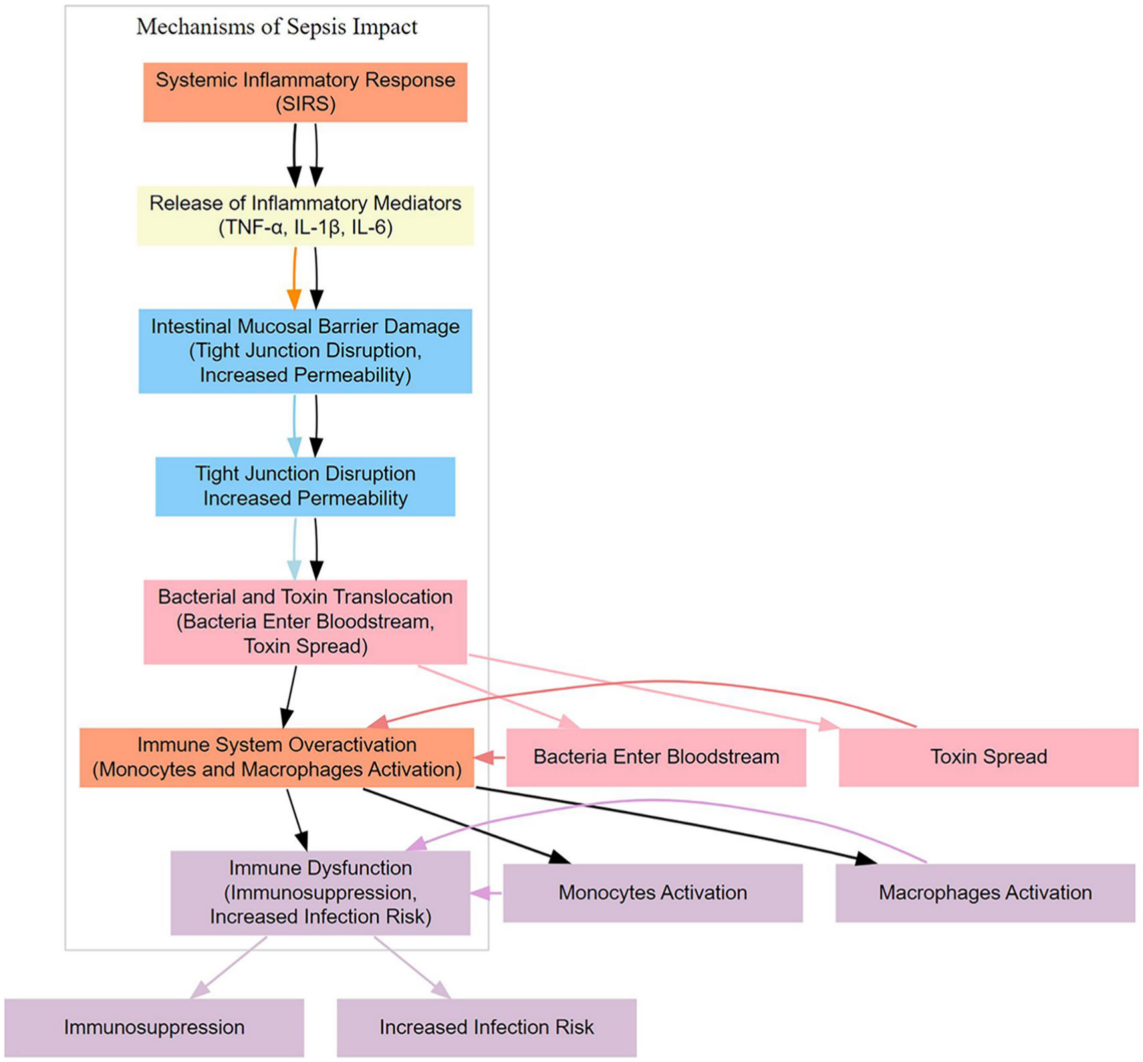
Mechanisms of sepsis impact.

The immune system plays a crucial role in the gut of sepsis patients, with its activities primarily influenced by the inflammatory response triggered by the infection source (8, 9) (Figure 1). The immune system is essential in maintaining intestinal homeostasis, preventing pathogen invasion, and protecting the integrity of the mucosal barrier.

After the onset of sepsis, the immune system is activated to respond to the pathogens and toxins released by the infection source. Inflammation is a natural response of the immune system aimed at clearing harmful substances, repairing damaged tissue, and preventing the spread of infection. However, in sepsis patients, the level of inflammation can become excessively heightened, leading to collateral damage to normal tissues by the immune system. Immune cells, such as leukocytes and macrophages, are recruited to the site of infection to clear pathogens. In the gut, this can result in the release of inflammatory mediators, including cytokines and other inflammatory agents, which, when released in excess, can negatively impact the intestinal mucosal barrier. The overactivation of the immune system may increase the permeability of the mucosal barrier, making it easier for bacteria, toxins, and other harmful substances to cross the mucosa. Simultaneously, the immune attack May damage normal tissues, contributing to the formation of ulcers. Therefore, the activity of the immune system in the gut of sepsis patients, particularly the excessive degree of inflammation, can adversely affect the integrity of the mucosal barrier, thereby worsening the condition (10). When managing sepsis, balancing the immune response to mitigate excessive inflammation and prevent damage to the gut is a critical therapeutic consideration.

## 3 Basic principles of enteral nutrition

### 3.1 Definition of enteral nutrition

Enteral nutrition refers to the delivery of nutrients into the body through oral or intestinal routes to meet the body’s nutritional requirements. This approach utilizes the absorptive and metabolic capacity of the intestines to provide essential nutrients necessary for maintaining life (11).

Key concepts of enteral nutrition include:

#### 3.1.1 Oral route

The most common form of enteral nutrition is through oral intake. This involves the consumption of various foods, beverages, or specialized nutritional supplements via the oral cavity. The oral route not only aligns with physiological habits but also provides the body with essential energy, proteins, vitamins, and minerals through the normal gastrointestinal absorption process.

#### 3.1.2 Intestinal route

When patients are unable to consume sufficient nutrients orally, the intestinal route serves as an alternative option. This can be achieved by delivering specially formulated nutrient solutions directly into the stomach or small intestine via a gastric or jejunal tube. This method bypasses oral intake and delivers nutrients directly, suitable for certain special circumstances such as postoperative recovery, critically ill patients, or individuals with swallowing difficulties.

#### 3.1.3 Absorption and utilization

The intestine is the primary site for nutrient absorption, where epithelial cells of the mucosa transport nutrients into the bloodstream through active and passive absorption mechanisms. Carbohydrates, fats, proteins, vitamins, minerals, and other nutrients are broken down, absorbed, and metabolized in the intestine to provide the body with energy and essential substances for maintaining physiological functions.

Providing nutrition via the enteral route contributes to maintaining the health and integrity of the intestinal mucosa and promotes normal intestinal function. This holds significant clinical significance for patients who cannot obtain sufficient nutrition through regular dietary intake, such as those undergoing postoperative recovery, experiencing malnutrition, or suffering from intestinal dysfunction.

### 3.2 Maintenance of intestinal mucosal barrier

#### 3.2.1 Provision of nutritional support

The intestine plays a crucial role in maintaining immune function and mucosal integrity. Enteral nutrition delivers various nutrients, including proteins, vitamins, and minerals, via oral or intestinal routes, providing the required energy and essential components to mucosal cells. This aids in promoting mucosal cell repair and regeneration, maintaining the normal structure and function of the intestine. Through the action of enteral nutrition, infusion of specific nutrients helps alleviate organ damage caused by sepsis (12–15).

#### 3.2.2 Anti-inflammatory effects

Certain components of enteral nutrition, such as Omega-3 fatty acids, antioxidants, glucose, and calcitriol, are known to possess anti-inflammatory properties (16–19). They can attenuate inflammatory responses through various pathways by regulating the release of inflammatory mediators, thereby reducing mucosal barrier damage (20).

#### 3.2.3 Support of the immune system

Enteral nutrition aids in maintaining the normal function of the immune system. Adequate nutritional support enhances the immune system’s resistance to infection and mitigates the inflammatory process. Maintaining immune system balance helps prevent excessive immune responses, thereby reducing damage to the intestinal mucosa (10, 21, 22).

#### 3.2.4 Maintenance of intestinal microbial balance

Alterations in gut microbiota influence the progression of sepsis in patients (23, 24). Enteral nutrition helps maintain intestinal microbial balance, including the quantity and types of beneficial bacteria within the intestine. Beneficial bacteria play a critical role in maintaining the integrity of the intestinal mucosal barrier and combating harmful microorganisms. By providing nutrients such as probiotics and prebiotics, enteral nutrition supports a healthy gut microbiota, preventing the overgrowth of harmful bacteria and reducing potential inflammatory factors (25).

In summary, enteral nutrition plays a vital role in maintaining the intestinal mucosal barrier and attenuating the inflammatory process by providing the necessary nutrients for mucosal health, exerting anti-inflammatory effects, supporting the immune system, and maintaining intestinal microbial balance (6). This maintenance role holds significant clinical significance for many disease states, particularly inflammatory conditions related to intestinal health.

## 4 Nutritional status assessment in sepsis patients

### 4.1 Common nutritional issues in sepsis patients

#### 4.1.1 Protein depletion

Sepsis patients often face significant protein depletion due to intense inflammatory responses and accelerated metabolism. Proteins are utilized in sepsis for synthesis of immune cells, tissue repair, and cell proliferation among various physiological processes. Consequently, protein depletion in sepsis May lead to muscle loss, compromised immune function, and associated nutritional problems.

#### 4.1.2 Imbalance of trace elements

Infection and inflammatory processes may lead to imbalances in trace elements within the body. For instance, elements like zinc, iron, and selenium might be utilized extensively under inflammatory conditions, potentially depleting reserves in the body. This depletion could impair immune function and disrupt cellular metabolism.

#### 4.1.3 Abnormal energy metabolism

Sepsis patients typically exhibit heightened metabolic activity, resulting in significantly increased energy demands. Inadequate energy supply may lead to abnormal energy metabolism, resulting in weight loss, muscle wasting, and related issues.

#### 4.1.4 Vitamin deficiency

Chronic inflammatory states may cause metabolic abnormalities in vitamins, particularly vitamins C and D. Vitamins play crucial roles in immune function and tissue repair, hence deficiencies may further impact patient recovery.

#### 4.1.5 Fluid and electrolyte imbalance

Sepsis patients may experience significant fluid loss and electrolyte disturbances, especially during fever, vomiting, diarrhea, and other infection-induced conditions. This may lead to reduced blood volume, impaired circulatory function, and subsequently affect normal organ function.

Therefore, maintaining an appropriate nutritional status is crucial when managing sepsis patients. Individualized nutritional support plans, including adequate protein intake, supplementation of trace elements, appropriate energy provision, and balancing of vitamins and electrolytes, are essential for promoting patient recovery and mitigating the inflammatory process.

### 4.2 The necessity of nutritional support

Sepsis, a severe infectious disease, typically accompanies intense immune responses and systemic inflammation. In this pathological state, patients undergo a series of complex physiological changes, including heightened metabolic activity, excessive activation of the immune system, and increased demand for tissue repair. These physiological changes pose significant challenges to patients’ nutritional requirements, rendering traditional diets insufficient to meet their body’s needs. Therefore, providing specialized nutritional support is crucial for sepsis patients. Firstly, sepsis patients often face rapid protein depletion and significant loss. Proteins are critical components for tissue repair, immune responses, and cell proliferation. Hence, sepsis patients require increased protein intake to meet these additional demands (26). Secondly, energy requirements are markedly elevated during sepsis due to excessive metabolic activity and elevated body temperature. Normal diets may not provide sufficient energy; thus, specialized high-energy nutritional support, such as high-energy oral supplements or intravenous infusion via enteral routes, may be beneficial. Moreover, the loss of trace elements, vitamins, and electrolytes is another reason sepsis patients require specialized nutritional support. These substances play crucial roles in immune function, anti-inflammatory responses, and tissue repair.

In summary, due to their unique physiological status, sepsis patients require specialized nutritional support to ensure adequate intake of protein, energy, and other essential nutrients. This support facilitates recovery, alleviates inflammation, and ultimately improves patient survival and quality of life. Therefore, individualized and comprehensive nutritional support plans are indispensable components of sepsis patient management.

## 5 Clinical research and advancements

Recent research has deeply explored the crucial role of enteral nutrition in the treatment of sepsis, particularly regarding changes in the gut microbiota (27). In the realm of personalized nutrition, researchers are focusing on tailoring enteral nutrition regimens based on the patient’s pathophysiological conditions and metabolic needs. By analyzing the patient’s microbiome, they are proposing new approaches to adjust formulations, aiming to improve immune regulation and reduce inflammation to optimize treatment outcomes (Table 1).

**Table 1:** Beneficial nutrients in enteral nutrition for sepsis: evidence from recent studies.

Study	Nutrient	Dates	Subjects	Outcomes
Ezzeldin Saleh (16)	Omega-3	2018	Septic patients	Improved organ function and reduced ICU stay in septic patients.
Huanqin et al. (34)	Probiotics	2019–2022	Elderly patients with sepsis	Significantly improved intestinal function, nutritional status, and prognosis.
Yeh et al. (18, 19)	Vitamin D	2022	CLP mice	Reduced intestinal inflammation and improved intestinal epithelial integrity.
Juliette et al. (22)	Citrulline	2022	CLP mice	Enhanced immune function and reduced secondary infections.
Jiabao et al. (13, 14)	Octanoic acid	2023	Septic rats	Alleviated acute liver injury in septic rats.
Tomohiro and Ippei (35)	Low-methoxyl Pectin	2023	Septic rats	Reduced risk of diarrhea and decreased local inflammation.

At the molecular level, recent research has concentrated on uncovering the mechanisms by which enteral nutrition regulates immune cell activity. Studies on specific nutritional components suggest that they may modulate immune cell functions by affecting various signaling pathways and gene expression (28). For instance, enteral nutrition has been shown to significantly alleviate hypercatabolism in endotoxemic rats through the ghrelin/GHS-R1α-POMC pathway (29). Supplementation with citrulline can markedly improve B cell suppression and plasma cell differentiation in sepsis, reducing immunosuppression and secondary infections (22). Similarly, in rat models, it was found that the addition of caprylic acid to enteral nutrition, compared to enteral nutrition alone, alleviated acute liver injury and improved gut function in septic rats by activating the PPARγ/STAT-1/MyD88 pathway (13, 14). The targeted inclusion of specific nutrients in enteral nutrition therapy holds promise for modulating inflammatory responses, providing a new theoretical foundation for designing more precise immunomodulatory strategies.

Recent clinical studies have found that early enteral nutrition can improve the severity of disease in postoperative sepsis patients (30). Similarly, early enteral nutrition may offer potential benefits for sepsis patients with concomitant muscle wasting and those with circulatory shock (31, 32). It may be particularly beneficial for sepsis patients with lower lactate levels (33). For elderly sepsis patients, early administration of probiotic-enriched enteral nutrition has been shown to significantly enhance gut function, nutritional status, and prognosis (34). Dietary additions such as low-methoxy pectin can effectively reduce the risk of diarrhea and alleviate local inflammation in sepsis conditions (35). Additionally, enteral nutrition containing dietary fiber has demonstrated substantial potential in reducing sepsis-related outcomes and preventing the development of sepsis in critically ill patients (15). However, in critically ill patients requiring vasopressors, enteral nutrition should be delayed or cautiously administered if cardiac output is low and dobutamine is needed, or in cases of high SAPS II scores with multiple organ failure (36). Early moderate enteral feeding (targeting 60% of requirements) can improve gut barrier function and nutritional and inflammatory status without increasing the incidence of feeding intolerance symptoms in sepsis (37). While early enteral nutrition helps maintain gut barrier function, it may pose risks of complications such as intestinal ischemia in patients with septic shock. Recent research suggests that a “small and better” enteral nutrition strategy during the acute phase may be safer, taking into account the severity of illness, dosage of vasopressors, and nutritional needs (38).

Evaluating the effectiveness of enteral nutrition during treatment is crucial (39, 40). Concurrently, early prediction and prevention of complications related to enteral nutrition remain a clinical focus. Research has found that a higher ratio of Firmicutes to Bacteroidetes and greater microbial diversity on the first day of enteral nutrition may aid in early prediction of enteral nutrition tolerance (41). The establishment and validation of XGBoost models can be used for early prediction of enteral nutrition initiation in ICU patients (42). New predictive models based on deep learning effectively forecast enteral feeding intolerance in sepsis patients in the ICU, which can be used for stratifying the risk of enteral nutrition intolerance in sepsis patients (43). The predictive capability of norepinephrine equivalent dose (NEQ) and the mean arterial pressure (MAP)/NEQ index can distinguish earlier whether shock patients receiving vasopressors are suitable for starting enteral nutrition, thus reducing the incidence of feeding intolerance and non-occlusive mesenteric ischemia (44, 45). Studies have shown that using improved ultrasound methods to guide enteral nutrition in sepsis patients allows for faster initiation of nutritional support and better outcomes compared to traditional clinical experience (40). For septic shock patients, a comprehensive assessment based on the clinical condition is required to determine the suitability for enteral nutrition. Obese patients are more prone to enteral nutrition intolerance, and the dosage of norepinephrine is significantly associated with tolerance (46).

Recent studies indicate that early enteral nutrition may be beneficial for sepsis patients (47–52). These latest findings offer valuable insights into the role of enteral nutrition in sepsis treatment and open new avenues for personalized treatment and innovative therapies. However, further research is needed to comprehensively understand the best practices and application strategies for enteral nutrition in sepsis treatment.

## 6 Limitations and challenges

### 6.1 Potential risks

#### 6.1.1 Risk of infection

Enteral or tube feeding may pose a risk of infection, particularly when improper procedures are followed during tube insertion or maintenance. Bacteria can enter the gastrointestinal tract through the tube, increasing the likelihood of infection. Regular monitoring of tube placement, maintaining cleanliness of the tube, and employing sterile techniques are vital measures for reducing the risk of infection.

#### 6.1.2 Risk of hypersensitivity reactions

In certain cases, patients may develop allergies or hypersensitivity reactions to certain components of enteral nutrition. This may include adverse reactions to specific proteins, lipid emulsions, or other additives. Therefore, when devising enteral nutrition regimens, careful consideration of the patient’s allergy history and individual differences is warranted.

#### 6.1.3 Electrolyte imbalance

Prolonged enteral nutrition can lead to electrolyte imbalances, especially in patients with intestinal absorption issues. This may involve loss or accumulation of electrolytes such as sodium, potassium, calcium, affecting the function of the cardiovascular and nervous systems.

#### 6.1.4 Gastrointestinal complications

The insertion of gastrointestinal tubes may result in complications such as gastric perforation, tube dislocation, among others. These complications could increase patient discomfort and treatment complexity (53, 54).

### 6.2 Therapeutic challenges

#### 6.2.1 Patient acceptance

Some patients may exhibit psychological or physiological aversions to enteral or tube feeding, impacting treatment adherence. Additionally, septic patients may experience symptoms like anorexia and vomiting, reducing the feasibility of oral intake or enteral nutrition.

#### 6.2.2 Variability in nutritional requirements

The progression and treatment of sepsis can alter patients’ nutritional needs. For instance, infection and inflammation states may accelerate metabolism, increasing the demand for protein and energy. Hence, nutritional regimens need timely adjustments to meet the evolving needs of patients.

#### 6.2.3 Impaired intestinal function

Some septic patients may suffer from impaired intestinal function, affecting the absorption of enteral nutrition. This might necessitate the selection of more easily absorbable special formulas or consideration of alternative routes of nutrition support, such as parenteral nutrition.

In conclusion, understanding and addressing the potential risks and therapeutic challenges of enteral nutrition in the treatment of sepsis are crucial for enhancing treatment efficacy. Individualized treatment plans, close monitoring, and timely adjustments are key strategies for addressing these issues.

## 7 Key strategies for treatment

### 7.1 Key strategies for enteral nutrition in sepsis patients

#### 7.1.1 Multifaceted consideration for individualized treatment plans

When devising personalized enteral nutrition regimens, a comprehensive assessment of the patient’s physical condition, nutritional needs, metabolic status, and treatment goals is necessary. This may involve utilizing advanced nutritional assessment tools and medical techniques to ensure the accuracy and effectiveness of the plan.

#### 7.1.2 Precision management through monitoring and adjustment

Close monitoring forms the cornerstone of successful treatment. Regular assessment of the patient’s nutritional indicators, physiological parameters, and clinical presentation aids in timely detection of issues and subsequent adjustments. This necessitates healthcare professionals to possess a high level of expertise and skills to ensure the health and safety of patients.

#### 7.1.3 Optimization of nutritional support and immune modulation

Nutritional support should not only aim to meet energy and nutrient requirements but also focus on its role in immune function modulation. Therefore, the design of enteral nutrition regimens should consider the types, proportions, and intake levels of nutrients to maximize the normal functioning of the immune system.

#### 7.1.4 Delicate regulation of microbial balance

The gut microbiota play a significant role in the treatment of sepsis. Hence, the formulation of enteral nutrition plans should consider the patient’s microbial characteristics and take measures to promote the growth of beneficial bacteria while inhibiting the overgrowth of pathogenic bacteria to maintain gut health.

#### 7.1.5 Comprehensive prevention and control of complications

Various complications may accompany the treatment of sepsis, such as malnutrition, infections, and organ dysfunction. Therefore, when formulating enteral nutrition plans, it is necessary to consider various potential complication risks and implement corresponding preventive and management measures to ensure smooth treatment progress.

During the process of providing enteral nutrition to sepsis patients, interdisciplinary teamwork among healthcare professionals is crucial. Only through comprehensive and meticulous assessment and management can treatment efficacy be maximized, laying a solid foundation for patient recovery and rehabilitation.

## 8 Summary and outlook

In summary, enteral nutrition holds promising prospects for the treatment of sepsis, offering a novel approach to improving patient outcomes. However, further research is warranted to gain a deeper understanding of the mechanisms underlying enteral nutrition and to develop more precise therapeutic strategies. These efforts aim to maximize both the survival rates and the quality of life for septic patients.
